# Supplementary material for: Causal Relationship between Aging and Anorexia Nervosa: A White-Matter-Microstructure-Mediated Mendelian Randomization Analysis
Source: Biomedicines. 2024 Aug 16;12(8):1874. doi: 10.3390/biomedicines12081874 (PMC11351342; doi:10.3390/biomedicines12081874)
Supplement: Supplementary file 1 [file biomedicines-12-01874-s001.zip › Supplementary Figure/Figure S4/Singe SNP plot of MR analysis for TL to ubm-b-1910.pdf]

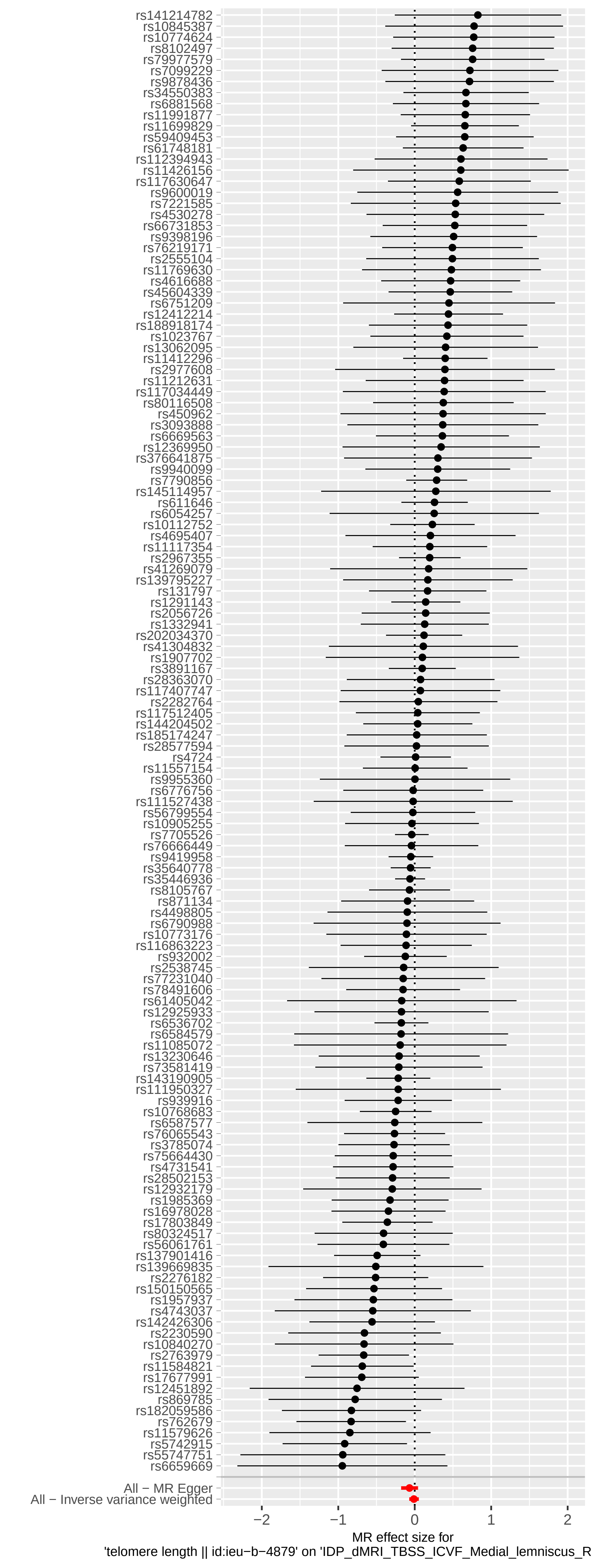
rs141214782
rs10845387
rs10774624
rs8102497
rs79977579
rs7099229
rs9878436
rs34550383
rs6881568
rs11991877
rs11699829
rs59409453
rs61748181
rs112394943
rs11426156
rs117630647
rs9600019
rs7221585
rs4530278
rs66731853
rs9398196
rs76219171
rs2555104
rs11769630
rs4616688
rs45604339
rs6751209
rs12412214
rs188918174
rs1023767
rs13062095
rs11412296
rs2977608
rs11212631
rs117034449
rs80116508
rs450962
rs3093888
rs6669563
rs12369950
rs376641875
rs9940099
rs7790856
rs145114957
rs611646
rs6054257
rs10112752
rs4695407
rs11117354
rs2967355
rs41269079
rs139795227
rs131797
rs1291143
rs2056726
rs1332941
rs202034370
rs41304832
rs1907702
rs3891167
rs28363070
rs117407747
rs2282764
rs117512405
rs144204502
rs185174247
rs28577594
rs4724
rs11557154
rs9955360
rs6776756
rs111527438
rs56799554
rs10905255
rs7705526
rs76666449
rs9419958
rs35640778
rs35446936
rs8105767
rs871134
rs4498805
rs6790988
rs10773176
rs116863223
rs932002
rs2538745
rs77231040
rs78491606
rs61405042
rs12925933
rs6536702
rs6584579
rs11085072
rs13230646
rs73581419
rs143190905
rs111950327
rs939916
rs10768683
rs6587577
rs76065543
rs3785074
rs75664430
rs4731541
rs28502153
rs12932179
rs1985369
rs16978028
rs17803849
rs80324517
rs56061761
rs137901416
rs139669835
rs2276182
rs150150565
rs1957937
rs4743037
rs142426306
rs2230590
rs10840270
rs2763979
rs11584821
rs17677991
rs12451892
rs869785
rs182059586
rs762679
rs11579626
rs5742915
rs55747751
rs6659669
All − MR Egger
All − Inverse variance weighted
−2
−1
0
1
2
MR effect size for
'telomere length || id:ieu−b−4879' on 'IDP_dMRI_TBSS_ICVF_Medial_lemniscus_R
